# Signatures of selection in core and accessory genomes indicate different ecological drivers of diversification among *Bacillus cereus* clades

**DOI:** 10.1111/mec.16490

**Published:** 2022-05-17

**Authors:** Hugh White, Michiel Vos, Samuel K. Sheppard, Ben Pascoe, Ben Raymond

**Affiliations:** ^1^ Centre for Ecology and Conservation University of Exeter Penryn UK; ^2^ European Centre for Environment and Human Health University of Exeter Medical School Environment and Sustainability Institute Penryn Campus UK; ^3^ Milner Centre for Evolution Department of Biology & Biotechnology University of Bath Bath UK

**Keywords:** *Bacillus cereus sensu lato*, comparative genomics, evolutionary genomics, horizontal gene transfer, niche differentiation, psychrotolerance

## Abstract

Bacterial clades are often ecologically distinct, despite extensive horizontal gene transfer (HGT). How selection works on different parts of bacterial pan‐genomes to drive and maintain the emergence of clades is unclear. Focusing on the three largest clades in the diverse and well‐studied *Bacillus cereus sensu lato* group, we identified clade‐specific core genes (present in all clade members) and then used clade‐specific allelic diversity to identify genes under purifying and diversifying selection. Clade‐specific accessory genes (present in a subset of strains within a clade) were characterized as being under selection using presence/absence in specific clades. Gene ontology analyses of genes under selection revealed that different gene functions were enriched in different clades. Furthermore, some gene functions were enriched only amongst clade‐specific core or accessory genomes. Genes under purifying selection were often clade‐specific, while genes under diversifying selection showed signs of frequent HGT. These patterns are consistent with different selection pressures acting on both the core and the accessory genomes of different clades and can lead to ecological divergence in both cases. Examining variation in allelic diversity allows us to uncover genes under clade‐specific selection, allowing ready identification of strains and their ecological niche.

## 1 INTRODUCTION

Bacterial strains often appear grouped together in distinct phylogenetic clusters, or “clades,” despite frequent homogenizing horizontal gene transfer (HGT; Buckee et al., 2008; Fraser et al., 2007; Schloss & Handelsman, 2004). Although uncovered by methods that are blind to ecology (Carroll et al., 2020; Guinebretière et al., 2008; Priest et al., 2004), these clades are often ecologically distinct from each other, both in phenotype and in genome content (Cohan, 2016; Hanage et al., 2006). How distinct bacterial phylogenetic clades appear is not fully understood (Doolittle & Papke, 2006). A key question in the debate is whether ecological differentiation is determined primarily by selection on the core genome (genes shared by all strains within a clade) or the accessory genome (genes shared only by a subset of those strains; Maistrenko et al., 2020; McInerney et al., 2017; Tettelin et al., 2008).

Selection in bacteria can be divided into three main categories: purifying selection, which removes deleterious alleles from a population; diversifying selection, which increases allelic diversity when rare alleles confer an advantage (McNally et al., 2019; Molina & Van Nimwegen, 2008); and directional selection, where alleles of genes are replaced by fitter variants (Cohan, 2016). Because recognizing directional selection requires data from a large number of isolates over a substantial time period (Buckee et al., 2008; Chen et al., 2006; Lefébure & Stanhope, 2007), we will focus on purifying and diversifying selection in this study. Purifying selection is prevalent amongst microbes (McNally et al., 2019) and common amongst core genes, either because they are integral to cellular processes or vital for survival in a given habitat (Cohan, 2016, 2017). Purifying selection may maintain cohesion within a clade by purging diversity, isolating clades from each other and maintaining their distinctiveness (Cohan, 2011, 2016). Diversifying selection also plays a key role in microbial evolution by maintaining multiple allelic variants within a population, a pattern which is common in genes linked to host colonization, phage resistance, and responses to vaccines and antibiotics (Harrow et al., 2021; McNally et al., 2019).

Quantifying the relative impact of selection on bacterial divergence is challenging, as this is dependent upon grouping multiple strains together as a single species, which has proven more difficult for bacteria than for plants and animals (Robinson et al., 2017). In addition to difficulties in recognizing directional selection, identifying which regions in the core genome are under selection is challenging due to inconsistent selection across sites within a gene and over time, the ubiquity of negative selection across genomes and mutation rate heterogeneity (Chen et al., 2006; Fay & Wu, 2003; Zhang et al., 2005). In contrast, selection on accessory genes is more simply inferred using presence/absence (Méric, Mageiros, Pascoe, et al., 2018; Vasquez‐Rifo et al., 2019). In this study, we aimed to identify regions under strong selection while accounting for other factors that influence the rate of molecular evolution (Méric, Mageiros, Pascoe, et al., 2018). Genes with higher or lower allelic diversity compared to the genomic average are probably under diversifying and purifying selection respectively, so we combined our expectations of allelic diversity with a method used to infer differences in allelic diversity between genes (Cohan, 2016; Méric, Mageiros, Pensar, et al., 2018; Shea et al., 2011). We applied this method to closely related bacterial clades with distinct ecological niches which we hypothesize have undergone divergent selection pressures. This methodology allows us to uncover the effect of selection within bacterial core genomes and compare selective pressures acting on different bacterial clades.

The *Bacillus cereus* (*Bc*) group contains a number of species with clinical or industrial importance: *Bacillus anthracis* (*Ba*), the causative agent of anthrax (Turnbull, 2002); *Bacillus cereus sensu stricto*, a causative agent of food‐poisoning (Messelhäußer & Ehling‐Schulz, 2018); *Bacillus thuringiensis* (*Bt*), a group of specialized invertebrate pathogens widely exploited as biopesticides (Bravo et al., 2007); and *Bacillus mycoides* (*Bm*), a psychrotolerant species which incorporates the former *Bacillus weihenstephanensis* (Lechner et al., 1998; Liu et al., 2018). The *Bc* group has been well studied, and there is increasing evidence that different clades have distinct ecological niches (Manktelow et al., 2021; Zheng et al., 2017), making this group ideal for exploring the importance of niche‐specific selection in driving clade divergence. For instance, carriage of enterotoxins and insecticidal toxin genes is known to vary strongly between clades (Cardazzo et al., 2008; Méric, Mageiros, Pascoe, et al., 2018); clade also correlates with habitat, thermal niche and cytotoxicity (Guinebretière et al., 2008, 2010; Raymond et al., 2010). Thermal niches and clade also predict relative fitness at different temperatures and fitness in a model insect host (Manktelow et al., 2021) and are linked to differences in biogeographical distribution (Drewnowska et al., 2020).

The phylogenetic structure of the group is well established and recoverable when alignments of multiple housekeeping genes (multilocus sequence typing [MLST]) or of the entire core genome are used to create phylogenies (Méric, Mageiros, Pascoe, et al., 2018; Priest et al., 2004). However, the taxonomy of the *Bc* group is much disputed (Carroll et al., 2020; Helgason et al., 2000; Liu et al., 2015). Different authors subdivide the group based on different levels of genetic distinctiveness; consequently, the number of informally recognized clades ranges between five and seven (Guinebretière et al., 2008; Méric, Mageiros, Pascoe, et al., 2018). In this study we will use the five‐clade structure initially recovered by MLST (Raymond et al., 2010), as these groups are clearly separated by large phylogenetic distances. This is important to our methodology as selection leading to clade divergence should have occurred far in the past. The gene‐by‐gene approach used here—which relies on known loci across multiple genomes—means that our methods cannot identify recent directional selection that has occurred only within a subset of a given clade or new imports that do not belong to a recognized locus (Sheppard et al., 2012) and so has not been designed to identify “ecotypes” with recent evolutionary origins (Cohan, 2016). We will focus on clades 1, 2 and 3 in the *Bc* group, originally named the “*anthracis*,” “*kurstaki*” and “*weihenstephanensis*” clades respectively (Priest et al., 2004). While *Bacillus cereus sensu stricto* strains are found in all three clades (Patiño‐Navarrete & Sanchis, 2017), Clade 1 contains all *Ba* isolates, Clade 2 contains the majority of insecticidal *Bt* isolates while Clade 3 corresponds to the psychrotolerant *Bacillus mycoides* species (Liu et al., 2018). Bacteria in these clades are readily isolated from both clinical and natural environments and are well represented in genomic databases.

Here, we hypothesized that the three *Bc* clades are ecologically distinct due to selection on their core genomes. We predicted that different genes would be found within the clade‐specific core genomes of each clade, and that these genes would have different levels of allelic diversity in each clade, due to differences in selection pressure. We also hypothesized that ecological selection acts on the accessory genome and that HGT would be more frequent amongst diversifying genes, promoting diversification between clades. A large collection of *Bc* isolate genomes were used to reconstruct the five‐clade phylogeny identified in previous studies (Méric, Mageiros, Pascoe, et al., 2018; Priest et al., 2004). Based on comparisons of gene‐level allelic diversity to the *Bc* strict core genome average (Chattopadhyay et al., 2009; Méric, Mageiros, Pensar, et al., 2018), we identified genes core to each clade under selection, while presence/absence was used to identify accessory genes under selection (Méric, Mageiros, Pascoe, et al., 2018; Vasquez‐Rifo et al., 2019). These genes were subjected to Gene Ontology (GO) analyses to determine functional enrichment, while consistency indices (CInds) were used to estimate rates of HGT (Méric, Mageiros, Pensar, et al., 2018).

## 2 MATERIALS AND METHODS

### 2.1 Isolate selection

*Bacillus cereus* (*Bc*) sequence assemblies were gathered from the *Multispecies BIGSdb* database (Jolley & Maiden, 2010; https://sheppardlab.com/resources/). The isolates belonged to a recognized *Bc sl* species (Bazinet, 2017), were assembled from fewer than 3,000 contigs and had genome sizes in line with previous estimates for the group (Chun et al., 2012; Li et al., 2015; Méric, Mageiros, Pascoe, et al., 2018; Yi et al., 2016). In total, 352 isolate genomes met the selection criteria; of these, 24 isolates could not be assigned to clades with certainty and were removed from the analysis, leaving 328 isolate genomes (Table S1).

### 2.2 Creation of a reference pan‐genome

The assemblies were aligned using the mafft algorithm (Katoh & Standley, 2013) and a gene‐by‐gene approach. Assembly was conducted in the *BIGSdb* database (Sheppard et al., 2012). Contiguous sequences for each isolate were exported and entered into the Pan‐genome Iterative Refinement And Threshold Evaluation (pirate) toolbox (Bayliss et al., 2019). In the pirate toolbox, genome sequences are passed through multiple cluster thresholds to account for different selection strengths between isolates, avoiding over‐clustering and over‐splitting of groups (Bayliss et al., 2019). Sequences are filtered from input files and cd‐hit used to create sequence clusters. Markov Cluster (MCL) processes are repeated by pirate at default amino acid identity thresholds; the initial clustering at the lowest threshold identified “gene families” and continued until the highest user‐specified threshold. Unique MCL clusters at the highest threshold (95% amino acid identity) were classified as “unique alleles” (Bayliss et al., 2019). Paralogues were identified and loci were classified, then gene families with multiple loci were checked for over‐clustering. Genes were annotated using prokka (Seemann, 2014). pirate produced a gene presence/absence matrix, with each gene possessing its own identifier (Méric et al., 2014). The strict core genome for the entire data set was identified in excel by ordering genes based on the percentage of isolates within the group containing this gene. Genes were considered “strict core” if present in all isolates.

### 2.3 Phylogenetic analysis

A maximum‐likelihood phylogeny was produced using 1,004 “strict core” gene sequences. These strict core genes were present in all isolates used in this study. The concatenated sequences were aligned using mafft (Katoh & Standley, 2013). A maximum‐likelihood phylogeny (Gadagkar et al., 2005; Saitou & Imanishi, 1989) was produced using iq‐tree (Minh et al., 2020) with ModelFinder (Kalyaanamoorthy et al., 2017); the substitution model selected was GTR+F+R10. Inclusion of isolates assigned to clades in a previous study helped with clade recovery (Méric, Mageiros, Pascoe, et al., 2018). The tree was visualized using the r package *ggtree* (Yu et al., 2017).

### 2.4 Identifying core and accessory genes under selection within clades

To derive clade‐specific core and accessory genomes, strains within clades 1–3 were extracted in R by using *ggtree* (Yu et al., 2017). From these, we reconstructed clade‐specific core genomes consisting of genes present in ≥95% of the isolates within each clade. This led to a reduced chance of rejecting “clade‐defining” genes that have been lost in very derived isolates. Based on previous observations of allelic diversity and selection (Cohan, 2016; Dugatkin et al., 2005; Shea et al., 2011), genes of low allelic diversity were considered to be under purifying selection (i.e., selection leading to a reduced number of different alleles) while genes of high allelic diversity were considered to be under diversifying selection (i.e., selection leading to a greater number of alleles). All alleles of each gene in the strict core and clade‐specific core genomes were found through comparison of the isolates to a representative FASTA sequence in the *Multispecies BIGSdb Genome Comparator* under default parameters. Incomplete loci were ignored for pairwise comparison and paralogues were excluded entirely (Jolley & Maiden, 2010). We produced alignments using the mafft algorithm (Katoh & Standley, 2013). Diversity per locus was calculated for each gene by dividing the number of distinct alleles by the number of isolates containing that gene (Méric, Mageiros, Pascoe, et al., 2018; Méric, Mageiros, Pensar, et al., 2018). To distinguish selected regions from neutral ones while accounting for other factors influencing molecular evolution, the allelic diversity of each clade‐specific core gene was compared to the overall within‐clade diversity of the strict core genome (Fay & Wu, 2003; Méric, Mageiros, Pensar, et al., 2018). Those genes that lay outside two standard deviations of the core genome average (i.e., ~5% of the genes) were considered to have significantly low or high diversity and were therefore considered to be under selection (Cohan, 2016; Dugatkin et al., 2005; Shea et al., 2011). Clade‐specific accessory genes were defined as genes present in under 95% of a clade; gene presence/absence was used to identify accessory genes under selection as in previous studies (Méric, Mageiros, Pascoe, et al., 2018; Vasquez‐Rifo et al., 2019).

### 2.5 Gene Ontology analysis

To determine whether selected clade‐specific core and accessory genes were enriched for certain functions, each gene was assigned an identification number from the *Universal Protein Resource Knowledge Base* (UniProtKB; Boutet et al., 2007), based on pirate’s prediction of their gene name and function (Bayliss et al., 2019). *Bacillus subtilis* identification codes were used because the list of *B. subtilis* UniProtKB codes is more comprehensive than for the list for *Bc*, and gene names and functions are equivalent between the species. UniProtKB codes were also assigned for the strict core genes. Where a gene coded for a hypothetical function or had no suitable orthologue amongst *B. subtilis*, the gene was excluded from the analysis. Codes for each set of genes were entered into the Gene Enrichment Analysis tool on the *Gene Ontology* website, which uses the panther classification system (Mi et al., 2013). Over‐ and under‐representation of biological processes compared to the strict core genome was calculated using binomial testing (Rupert Jr, 2012) with replacement, approximating the hypergeometric distribution due to sample size (Rivals et al., 2007). A Bonferroni correction was used to account for multiple testing (Weisstein, 2004).

### 2.6 Inference of HGT using consistency indices

To examine the impact of HGT on clade formation, consistency indices (CInds) were used to estimate the level of HGT amongst genes under selection (Méric, Mageiros, Pensar, et al., 2018). CInds were created to detect homoplasy by comparing the fit of genetic alignment data to a phylogenetic tree. An alignment of allelic sequences from the same gene is compared to a reliable phylogeny produced using multiple conserved genes (Saitou & Imanishi, 1989) to produce a consistency index; lower indices indicate a greater degree of homoplasy. Homoplasy can be caused by independent mutation but is commonly assumed to be caused mainly by homologous recombination (Sanderson & Donoghue, 1989; Schliep, 2011), meaning that CInds can be used to infer levels of HGT within a group of bacterial strains.

Only genes that were present in all strains in the phylogeny and were considered under either purifying or diversifying selection in at least one clade were included in the consistency index analysis. Consistency indices were calculated for each gene using the r package *phangorn* (Schliep, 2011) and the maximum‐likelihood group phylogeny was used for comparisons. The process was repeated for all genes in the strict core genome (*n* = 1,004). The average CInd of each gene set was compared using a Wilcoxon–Mann–Whitney test. The frequency distribution of CInds for both gene sets was also examined. Both analyses have previously been conducted to test for significant differences in CInds between sets of genes (Méric, Mageiros, Pensar, et al., 2018).

## 3 RESULTS

### 3.1 The *Bacillus cereus* group phylogeny has a distinct clade structure

**FIGURE 1:**
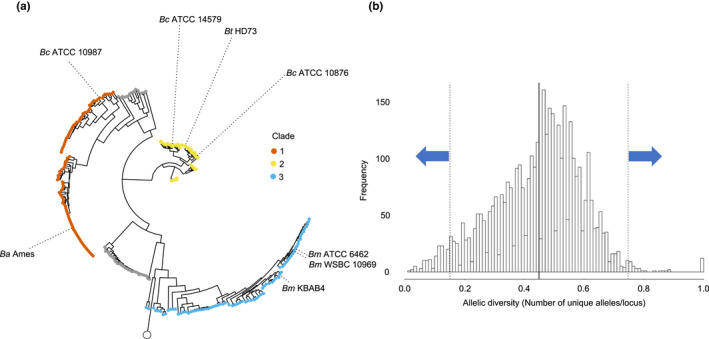
Identifying core and accessory genes under selection within the *Bacillus cereus sensu lato* (*Bc sl*) phylogeny. (a) Maximum‐likelihood phylogeny of the *Bc sl* group strains used in this study. The concatenated core genome sequences were aligned using mafft and fed into iq‐tree. Clade identity was determined through reference to type strains and consultation of existing clade metadata. (b) The process by which genes of low and high diversity were identified. The graph shows the frequency distribution of allelic diversity values across genes within a clade's flexible core genome. The solid line shows the mean diversity of the strict core genome within the clade and the dashed lines show the second standard deviation interval of the strict core genome

The strict core genome phylogeny divided *Bc* isolates into genetically distinct clades. In total, 328 genomes from the *Multispecies BIGSdb* database (Jolley & Maiden, 2010) met criteria for the study, with an average size of ~5.6 ± 0.3 Mb (Table S1) and an average contig number of 285. Variation in assembly sequence size and contig number was consistent with other published estimates of *Bacillus* group genome sizes (Chun et al., 2012; Li et al., 2015; Méric, Mageiros, Pascoe, et al., 2018; Takeno et al., 2012; Yi et al., 2016). The group pan‐genome produced by pirate contained 36,687 genes, consisting of 1,004 strict core genes excluding homologues and 35,679 accessory genes. A maximum‐likelihood tree was produced using the concatenated strict core genome sequences and was consistent with the five‐clade phylogeny proposed by previous studies (Méric, Mageiros, Pascoe, et al., 2018; Sorokin et al., 2006; Figure 1a). The three largest clades, clades 1–3, contained 94, 95 and 78 isolates respectively (Figure 1a).

### 3.2 Functional enrichment is dependent on clade and whether the genes are core or accessory

**FIGURE 2:**
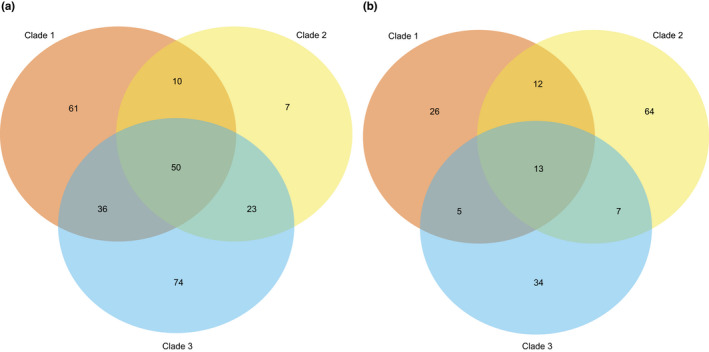
Venn diagram showing patterns of purifying and diversifying selection in the clade‐specific core genomes of the three largest *Bacillus cereus sensu lato* (*Bc sl*) clades. Numbers indicate the total number of genes that are experiencing selection (either purifying or diversifying), and location of numbers indicates whether the genes are experiencing selection in one clade or in multiple clades. (a) Genes under purifying selection (*n* = 261). (b) Genes under diversifying selection (*n* = 161)

Analysis of clade‐specific core genes under selection suggests different selective pressures acting on each *Bc* clade. Allelic diversity was calculated for each gene that was present in all strains within a specific clade—the clade‐specific core genes—and compared to the strict core genome average to identify genes under purifying or diversifying selection (Figure 1b). Out of 4,383 clade‐specific core genes across three clades, 261 had allelic diversity significantly lower than the within‐clade strict core genome average (two standard deviations below the mean), while 161 had significantly higher allelic diversity than the within‐clade strict core genome average (two standard deviations above the mean; Table S2). Despite some genes appearing in multiple clade‐specific core genomes, most genes were conserved or diverse only within one clade (Figure 2). Genes found to be conserved or diverse in previous studies were also found to be conserved or diverse respectively in this study. These included the *cspA* gene, coding for a highly conserved cold‐shock protein used to classify the psychrotolerant *Bm* (Lechner et al., 1998), and the *hag* gene which encodes a diverse bacterial flagella protein (Xu & Côté, 2006). Genes linked to functions such as protein export were conserved in all clades (Bost & Belin, 1997; Fröderberg et al., 2004; Table S2) and, as expected, Clade 3 contained many highly conserved cold‐shock proteins (Ermolenko & Makhatadze, 2002). Genes under diversifying selection in all clades included genes coding for flagellin (Xu & Côté, 2006) and the bacteriophage membrane receptor *yueB* (São‐José et al., 2004). A notable gene under diversifying selection in Clade 2 was *emrB*, a multidrug export protein (Lomovskaya & Lewis, 1992).

Clade‐specific accessory genes under selection were identified through presence/absence to a specific clade. In total, 5,239, 7,559 and 5,605 genes were found only in Clade 1, Clade 2 and Clade 3 respectively and present in less than 95% of the clade. Accessory genes under positive selection in each clade showed functions that are distinct to each clade. Of these, several are worthy of note; the Clade 1‐specific accessory genome included the gene *InlA*, which codes for internalin‐A and allows the invasion of mammal cells (Dhar et al., 2000), the Clade 2‐specific accessory genome included Cry toxins—key *Bt* insecticidal toxins—such as *cry2Ab* (Zheng et al., 2017), and the Clade 3‐specific accessory genome contained the gene *binA*, which produces a homologue to an insecticidal binary toxin component (Palma et al., 2014; Table S2).

### 3.3 GO analyses suggest clade‐specific selection acting on the core and accessory genomes of each *Bc* clade

**FIGURE 3:**
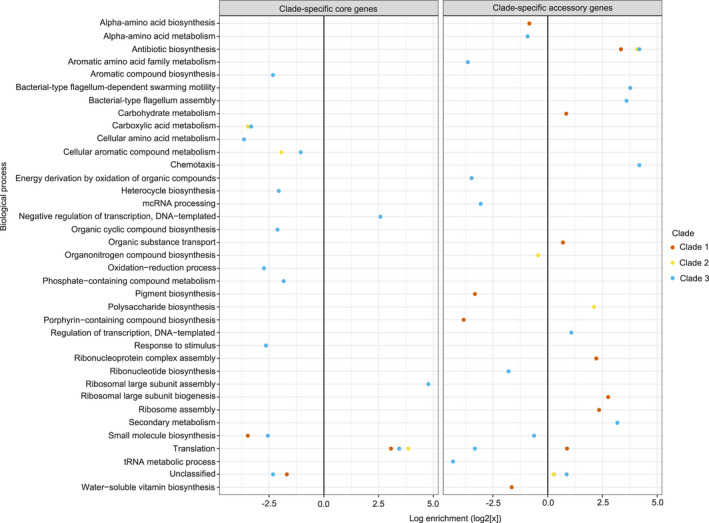
Significant enrichment of biological processes within clade‐specific conserved core genes and clade‐specific accessory genes across the major *Bacillus cereus sensu lato* (*Bc sl*) clades. Enrichment values were calculated using the Gene Ontology Enrichment analysis software available online, using a binomial test with Bonferroni correction. Only significant enrichments are shown

Binomial testing was used to measure the functional enrichment of biological processes (Ashburner et al., 2000; Gene Ontology Consortium, 2019) within clade‐specific core and accessory genomes (Mi et al., 2013) by comparison to the strict core genome. This methodology allowed ecological characterization of the clades and avoided a priori assumptions of relevance. Additionally, it avoids characterizing a clade by the possession of any one gene, as has often been the case in the *Bc sl* group (Bravo et al., 2007; Lechner et al., 1998). There was significant functional enrichment of biological processes amongst conserved and diverse clade‐specific core genes of all clades; conserved clade‐specific genes were often linked to translation (Figure 3a). However, some enrichment was clade‐specific: Clade 3 contained a greater number of conserved genes linked to negative regulation of transcription and fewer conserved genes linked to biosynthesis and stimulus response than would be expected based on the strict core genome (Figure 3a). The same was found to be the case for diverse clade‐specific genes; genes with uncharacterized functions were more common than expected within Clade 1 and less common than expected in Clades 2 and 3, but only Clade 2 showed unique functional enrichment, with more genes linked to antibiotic and antimicrobial resistance than expected. Functional enrichment of biological processes was robust when the criteria for considering genes under selection within a clade were relaxed to include ~10% of the clade‐specific core genomes as opposed to ~5% as described above.

Like the clade‐specific core genomes, there were disparities in functional enrichment between the clade‐specific accessory genomes. While some processes—such as antibiotic biosynthesis—were enriched in all clade‐specific accessory genomes, there were differences between the clades regarding the enrichment of other biological processes (Figure 3b). Interestingly, biological processes enriched within clade‐specific accessory genomes were not the same as those enriched within that clade's specific core genome. For instance, Clade 3 accessory genes were more likely to be linked to motility and secondary metabolism, while its clade‐specific core genome was not. Clade 3 was also not significantly enriched for accessory genes linked to negative regulation of transcription, while its core genome was (Figure 3b).

### 3.4 Genes under diversifying selection undergo more frequent HGT

**FIGURE 4:**
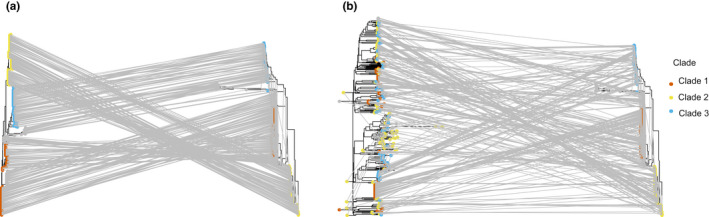
Consistency between phylogenies based on a single gene and a core genome. (a) Consistency between the conserved cold shock protein (*CspA*) gene phylogeny and the strict core genome (*n* = 1,004) of the *Bacillus cereus sensu lato* (*Bc sl*) group. A given isolate in each tree is connected to the same isolate in the other tree by a line. (b) Consistency between the diverse flagellin (*hag*) gene phylogeny and the strict core genome (*n* = 1,004) of the *Bc sl* group. A given isolate in each tree is connected to the same isolate in the other tree by a line

**FIGURE 5:**
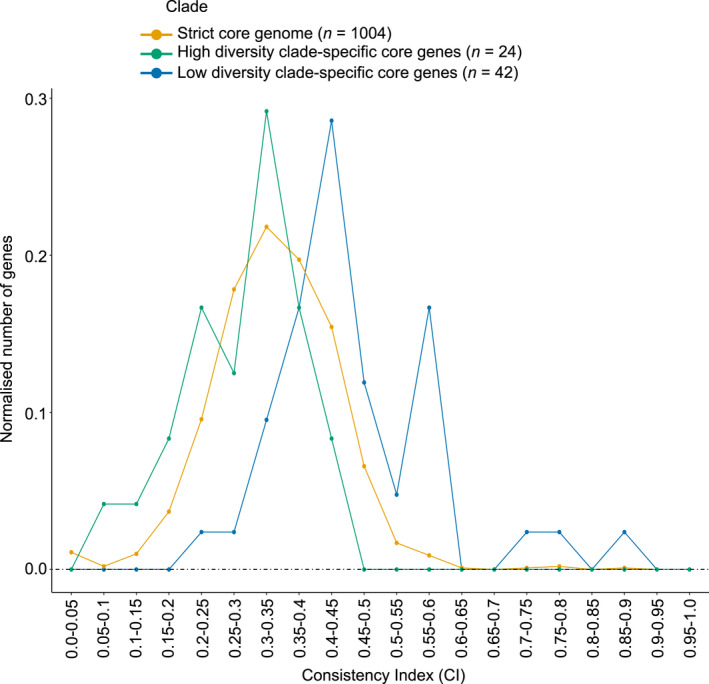
Consistency index distribution amongst genes under purifying selection (*n* = 42) and diversifying selection (*n* = 24). Consistency indices were calculated for each gene using the phangorn package in r and a maximum‐likelihood phylogeny was created using alignments of the concatenated core genome. The average consistency index of each gene set was compared to that of the strict core genome (*n* = 1,004). Normalized numbers of genes represent the number of genes with a given consistency index while controlling for the size of the data set

Two sets of clade‐specific core genes were suitable for CInd analysis; 42 genes with low allelic diversity and 24 genes with high allelic diversity were present in all 328 strains and therefore their gene phylogeny could be compared to the strict core genome phylogeny to check for inconsistencies that suggest HGT. CInds were calculated for clade‐specific core genes of high and low diversity, as well as for all genes in the strict core genome. The CInds of each gene set suggest that genes under diversifying selection undergo frequent HGT, while HGT is uncommon amongst conserved genes (Figure 4a,b); the mean CInd of conserved clade‐specific core genes (0.46 ± 0.02) was significantly higher than the mean of the strict core genome (0.34 ± 0.003; Wilcoxon–Mann–Whitney test; *U* = 33722, *p* = 4.435e^−11^). In contrast, the mean CInd of diverse clade‐specific core genes (0.28 ± 0.018) was significantly lower than for the strict core genome (Wilcoxon–Mann–Whitney test; *U* = 7893, *p* =.00385; Figure 5).

## 4 DISCUSSION

This study aimed to explore ecological differentiation between closely related bacterial clades and the role of selection in driving and maintaining this distinctiveness. To accomplish this, we tested bacterial genomes from an economically important and well‐studied model group for signatures of selection. The *Bc* group contains many different strains, all thought to be well‐adapted to exploit protein‐rich food such as cadavers (Manktelow et al., 2021; Rasigade et al., 2018). Despite high levels of genetic similarity, the clade structure of the group is distinct and robust to multiple phylogenetic methods. Clades have been associated with differences in fitness and virulence gene complement, as well as with distinct biogeographical and thermal niches (Cardazzo et al., 2008; Drewnowska et al., 2020; Guinebretière et al., 2008, 2010; Manktelow et al., 2021; Méric, Mageiros, Pascoe, et al., 2018; Zheng et al., 2017); here, we show that clade‐specific core and accessory genomes bear signatures consistent with niche‐specific selection.

We identified genes under putative purifying and diversifying selection within clade‐specific core genomes by comparison to diversity in the strict core genome. As mentioned, identifying genes undergoing selection presents computational and data sampling challenges (Buckee et al., 2008; Zhang et al., 2005); additionally, selection must be distinguished from other factors affecting allelic diversity (Chen et al., 2006; Fay & Wu, 2003; Zhang et al., 2005). This was achieved by using allelic diversity and comparison to the average genomic diversity to identify outliers under strong selection (Méric, Mageiros, Pensar, et al., 2018). Genes with very low or very high allelic diversity compared to the average are likely to be under strong purifying or diversifying selection (Cohan, 2016; Dugatkin et al., 2005; Shea et al., 2011). Amongst gene sets with non‐normally distributed allelic diversity values, using percentile values to encapsulate the most extreme 5% of the data would be suitable; however, due to a normal distribution of the data in this study, mean and *SD* filtering of allelic diversity provided a way to quickly identify genes under strong selection. It should be noted that low allelic diversity may occur due to purifying selection or due to directional selection combined with HGT (i.e., gene‐specific sweeps; Cohan, 2016); this may explain the low numbers of conserved genes within Clade 2. However, because the majority of conserved genes also showed low levels of HGT (Figure 5), we feel confident that the majority of conserved genes are the result of purifying selection; an in‐depth examination could identify genes from among these sets that are more likely to have undergone gene‐specific sweeps.

Analysis of clade‐specific conserved core genes suggested that core genes under purifying selection differed significantly between clades and supported previous hypotheses about the ecological distinctiveness of major *Bc* clades (Figure 3a). For example, consider our analysis of Clade 3, now recognized as *Bm* (Carroll et al., 2020). Here, the analysis of clade‐specific core genes identified the cold‐shock protein gene *cspA*, a unique sequence signature of which was used to originally classify the psychrotolerant *Bm* species (Lechner et al., 1998). Furthermore, Clade 3 possessed many conserved genes linked to ribosome assembly and negative regulation of transcription, and few linked to metabolism, biosynthetic processes and external stimuli responses (Ermolenko & Makhatadze, 2002). These features are characteristic of adaptation to low temperatures, where metabolic functions are downregulated in response to cold (Barria et al., 2013; López‐Maury et al., 2008; Tribelli & López, 2018). This supports other studies indicating that strains within Clade 3 are psychrotolerant specialists (Lechner et al., 1998; Liu et al., 2018; Manktelow et al., 2021) and demonstrates how the methodology used here can identify important genes with specific variants within ecologically distinct groups. Different patterns of enrichment amongst conserved clade‐specific core genes also suggest that the clades are ecologically distinct, and purifying selection may maintain new species by purging novel variation caused by mutation and HGT (Cohan, 2016, 2017).

We found evidence that diversifying selection within clade‐specific core genomes acts on different genes depending on the clade. While the *hag* flagellin gene was extremely diverse across all three clades, only genes of high allelic diversity within Clade 2 were enriched for functions linked to flagellum‐dependent motility. Flagellin is a common receptor for bacteriophages, and because variations in flagellin structure may prevent phage infection, this is a trait likely to be under diversifying selection (Nobrega et al., 2018). Clade 2 also has the largest proportion of isolates encoding insecticidal toxins and carries a greater number of insecticidal toxins than other clades (Méric, Mageiros, Pascoe, et al., 2018; Zheng et al., 2017). This supports the hypothesis that this clade is dominated by specialist insect pathogens (Raymond & Bonsall, 2013; Raymond & Federici, 2017; Raymond et al., 2010) and provides further evidence for the ecological distinctiveness of the clades.

Flagellar motility may also be important during the early stages of insect infection (Mazzantini et al., 2016); *Bt* mutants with reduced flagellar motility have reduced virulence when infecting larvae (Zhang et al., 1993). Diverse *Bt* genes were also more likely to be linked to antimicrobial resistance (Table S2). Antimicrobial resistance mechanisms are common in *Bc* strains (Abriouel et al., 2011; Bernhard et al., 1978) and are often under diversifying selection, which can result in the emergence and maintenance of allelic diversity for that trait (Levin, 1988; McNally et al., 2019). Diversifying selection on antibiotic resistance may be prevalent amongst Clade 2 strains because competition to enter insect cadavers first is intense (Garbutt et al., 2011; Van Leeuwen et al., 2015). Therefore, overcoming host defences and securing the first infection of a host may provide an advantage in pathogenic bacteria that is not seen in necrotrophic bacteria.

One of the aims of this study was to assess the importance of selection in maintaining bacterial species. Alternative drift‐based models of bacterial speciation assume that genetic differences between taxa are self‐reinforcing (Fraser et al., 2007). HGT can erode differences between neutrally diverging lineages and greater genetic distance leads to reduced HGT via a range of mechanisms (Fraser et al., 2007) There is evidence for these kinds of forces operating in the *Bc sl* group; for instance, HGT predominantly occurs within clades (Didelot et al., 2009). Nevertheless, one notable result of this study was the variation in inferred levels of HGT between loci under different forms of selection. Here, we used CInds to infer the prevalence of HGT. High CInds amongst conserved genes—such as the *cspA* gene—indicate low levels of HGT (Méric, Mageiros, Pensar, et al., 2018); in contrast, low CInds in diverse genes such as the *hag* gene imply high levels of HGT (Figures 4 and 5). At a fundamental level, all chromosomal genes undergo HGT at similar rates (Gogarten et al., 2002). However, the subsequent fate of horizontally transferred alleles differs depending on gene and gene function; this may be due to variation in selection strength and type between genes (Kivisaar, 2019; Nakamura et al., 2004). Our results indicate that the effects of HGT are strongly modulated by selection in the *Bc sl* group. When novel allelic diversity is favoured under diversifying selection, HGT can supply that diversity. However, purifying selection can also purge clade‐specific allelic variants that incur strong selective disadvantages in the “wrong” genetic background (Vos et al., 2015). Moderate levels of HGT therefore do not impede speciation, as seen in other species (Melendrez et al., 2016). Background levels of HGT are important, but selection can clearly act to promote clade identity and genetic coherence in the face of HGT.

While unlikely to be an issue in *Bc* due to intermediate levels of homologous recombination (Patiño‐Navarrete & Sanchis, 2017), CInds are probably most effective at identifying patterns of HGT when levels are low or intermediate; at high rates of HGT genes may be spread sufficiently widely so that genes received via HGT cannot be distinguished from genes received via linear descent (Andam & Gogarten, 2011; Sanderson & Donoghue, 1989). Spotting inconsistencies may also be difficult in conserved genes due to the small number of differences between genes. However, given levels of HGT are roughly intermediate for all gene sets (~0.5) and that conserved genes with small differences are sufficiently different to be used for reconstructing phylogenies (Saitou & Imanishi, 1989), these would seem to be minor concerns.

The role of accessory genomes in ecological specialization is widely accepted (Brockhurst et al., 2019; Cobo‐Simón & Tamames, 2017); *Bt*, which carries key virulence factors primarily on large plasmids, is a well‐known example (Zheng et al., 2017). As with the core genome analysis, accessory genes unique to each clade were significantly enriched for specific biological processes. Furthermore, the processes enriched within a clade‐specific core genome often differed from the processes enriched within the specific accessory genome of the same clade. For instance, the Clade 3 accessory genome was enriched for genes linked to motility and secondary metabolic processes, while its core genome was not. The utility of presence/absence for identifying accessory genes under selection is still debated, as strains accumulate a mix of deleterious, beneficial and neutral genes and the frequency of beneficial accessory genes is unclear (Vos & Eyre‐Walker, 2017). Despite this, presence/absence of specific accessory genes has been found to be biologically meaningful in other studies (Cohen et al., 2013; Méric, Mageiros, Pascoe, et al., 2018; Vasquez‐Rifo et al., 2019). With this considered, our results would suggest that both the core and accessory genome determine a strain's ecology.

While these results indicate the importance of chromosomal core and accessory genes to strain ecology, they should be taken with caution for three reasons. First, enrichment within the accessory genome may not be representative of all strains within a clade; the majority of genes in bacterial pan‐genomes are either common (“core” or nearly core) or extremely rare (accessory; Haegeman & Weitz, 2012). Because the *Bc sl* clades consist of isolates assigned to different species or ecotypes—for instance, both Clades 1 and 2 contain strains identified as *Bt* (Méric, Mageiros, Pascoe, et al., 2018)—the enrichment of certain biological processes within a clade's accessory genome may be due to high numbers of rare genes that are possessed by a minority of the clade in question. Second, the different functional enrichment in clade‐specific core and accessory genomes may reflect differences in selection over time as opposed to differences in function; within one species of bacterium, accessory gene content change occurs at faster rates but is retained less readily than amino acid substitution in the core genome (Wielgoss et al., 2016), implying that accessory genomes reflect current selection and core genomes reflect past selection. Third, this study did not attempt to incorporate plasmid sequences into the analysis. While it was not possible to differentiate between chromosomal and plasmid DNA in all isolates, we did not explicitly analyse plasmid sequences in this study. While some plasmids are stably associated with *Bc* lineages and therefore considered part of the “core genome” (Méric, Mageiros, Pascoe, et al., 2018; Zheng et al., 2017), many plasmids are highly mobile and carry genes encoding several key virulence traits (Patiño‐Navarrete & Sanchis, 2017; Schnepf et al., 1998). While analysis of the selection pressures that formed the *Bc* clades will benefit by excluding plasmid sequences (by reducing the confounding effect that highly mobile plasmids may have on analysis), future researchers may wish to incorporate these important parts of the *Bc sl* pan‐genome. Therefore, future iterations of this methodology may benefit from two modifications: splitting analysis of the accessory genome into genes of intermediate and low frequency within a clade (Inglin et al., 2018) and the incorporation of plasmid sequence data.

It is interesting that Clade 1 does not appear to possess any significant clade‐specific enrichments, aside from deficiencies in certain biosynthetic processes (Figure 3b) and the possession of the internalin‐A protein gene *inlA* (allowing for epithelial cell invasion) in its clade‐specific core genome (Dhar et al., 2000). We hypothesized that the *anthracis* clade would consist of necromenic (cadaver‐associated) bacteria that may specialize on vertebrates (Manktelow et al., 2021) although *Ba* itself is a clonal expansion and represents only a small part of the diversity in this group. Clade 1 includes at least six currently recognized species, though one proposed revision suggests lumping all these groups into a single taxon based on a 92.5% average nucleotide identify (ANI; Carroll et al., 2020). Regardless of current taxonomic disputes, the clade splits into two groups separated by a 94% ANI. These two branches of Clade 1 were previously described as *PanC* Groups II and III, corresponding to *Bacillus paranthracis* and allies and *Bacillus albus*/*wiedmannii* and allies respectively (Guinebretière et al., 2008, 2010). There is evidence for differences in phenotype and biogeography between these groups (Drewnowska et al., 2020; Guinebretière et al., 2008, 2010). “Lumping” these groups into a single clade may be obscuring ecological distinctiveness in Clade 1. While useful for identifying the selection pressures that formed the *Bc* clades and that are currently creating diversity within each clade, our results should not be taken to mean that the clades are ecologically monolithic. Repeating this analysis using the seven‐clade phylogeny of Guinebretière et al. (2008) and with greater representation in these subgroups may reveal ecological distinctions that were not seen in this study.

This possibility suggests how this selection‐informed analysis may be used for refining taxonomic decision‐making. Methods based on raw genetic differences, such as ANI, appear highly objective; however, decisions still need to be made on how to apply rules and what level of differentiation is appropriate for describing species in a particular group (Carroll et al., 2020; Vos, 2011). There are advantages in describing species as units with real ecological and phenotypic distinctiveness; if groups recognized by ANI‐based decisions also show coherent patterns of selection, it provides another means of assessing whether a species definition is of practical value. Another pragmatic application of genome‐wide analysis of conserved genes is its value in identifying key ecological traits and single loci that can be used for species‐level identification; one example from this study is the wealth of psychrotolerance traits found in Clade 3, exemplified by the conserved cold‐shock gene *cspA*.

In conclusion, this study showed that functional enrichment in both core and accessory genes is heavily dependent on clade in the *Bc* bacterial group. Key ecological traits associated with *Bacillus* species—such as antimicrobial and insecticidal activity in *Bt* strains and psychrotolerance in *Bm* strains—were among those enriched in specific clades, supporting the hypothesis that clades within the group formed due to different selection pressures and have distinct ecologies. The core and accessory genomes of each clade appear to experience selection on different traits, highlighting the importance of considering both when determining clade ecology. High levels of HGT amongst diversifying core genes suggest that HGT plays a key role in promoting diversification within the *Bc sl* group. Lastly, this analysis identified genes, such as the *cspA* gene in Clade 3, that can be used to identify strains to the clade level and to infer their ecological niche, allowing easier determination of strains’ potential to harm humans and to act as biopesticides, with the commensurate benefits to agricultural and medical practices.

## AUTHOR CONTRIBUTIONS

H.W. designed the study, performed research, analysed data and was the primary writer for the manuscript. S.K.S. and B.R. helped design the study, and S.K.S. contributed the use of pirate and the *BIGSdb* database. S.K.S., B.R. and M.V. all contributed to the writing of the manuscript.

## CONFLICT OF INTEREST

The authors declare no competing interests.

### OPEN RESEARCH BADGES

This article has earned an Open Data Badge for making publicly available the digitally‐shareable data necessary to reproduce the reported results. The data is available at https://doi.org/10.24378/exe.3992, https://hdl.handle.net/10871/129565.

## Supporting information

Table S1‐S3

## Data Availability

Genetic data can be accessed from public databases by referring to the strain accession numbers in Table S1. Sample metadata are available from the *Multispecies BIGSdb* (Jolley & Maiden, 2010; https://sheppardlab.com/resources/) and are available in Table S1. Metadata include *Multispecies BIGSdb* ID, the clade the strain was assigned to in this study, isolate identifier, aliases, pathotype, species source, lineage, serovar, clinical isolate, sequence length (bp) and accession number. The pirate Pipeline is available through GitHub (https://github.com/SionBayliss/PIRATE ). Details of the clade‐specific core genes that showed extremely high and low allelic diversity can be found in Table S2a,b. Details of clade‐specific accessory genes can also be found in Table S2c. UniprotKB codes are available for each gene from the UniProt Knowledgebase (UniProtKB; https://www.uniprot.org/) and are listed next to their respective gene in Table S2. Metadata include pirate ID number, the clades in which a gene was conserved/diverse/accessory, consensus gene name, consensus gene product and UniProtKB code. Raw output from the pirate pipeline (both the excel summary and the identified “gene family”.FASTA files), the maximum‐likelihood tree file and iq‐tree command lines, output from the Gene Ontology analysis tool, and the raw output from analysis of consistency indices will be made available publicly through Open Research Exeter (ORE; https://ore.exeter.ac.uk/repository/handle/10036/10890) upon acceptance and publication. The iq‐tree and *R* scripts used to generate relevant output (maximum‐likelihood phylogeny Figure 1a, Gene Ontology graph Figure 3 and consistency index graph Figure 5) will also be stored here.
